# Novel CircRNAs in Hub ceRNA Axis Regulate Gastric Cancer Prognosis and Microenvironment

**DOI:** 10.3389/fmed.2021.771206

**Published:** 2021-11-08

**Authors:** Xianghui Li, Zhiyan Li, Ping Liu, Shichao Ai, Feng Sun, Qiongyuan Hu, Yuxiang Dong, Xuefeng Xia, Wenxian Guan, Song Liu

**Affiliations:** ^1^Department of Gastrointestinal Surgery, Affiliated Drum Tower Hospital, Medical School of Nanjing University, Nanjing, China; ^2^Wuxi People's Hospital Affiliated to Nanjing Medical University, Wuxi, China; ^3^First Clinical Medical College of Nanjing Medical University, Nanjing, China

**Keywords:** gastric cancer, Immunotherapy, novel circular RNA, ceRNA axis, immunosuppressive microenvironment

## Abstract

Gastric cancer (GC) is one of the most prevalent malignancies with an unfavorable survival rate. Immunotherapy may contribute to a better prognosis. However, several phase III trials failed. Circular RNA (circRNA) is a novel type of non-coding RNA, plays a vital role in the progression of tumors. The expression and function of circRNA in the GC immune microenvironment remain obscure. In this study, we utilized a bioinformatic analysis to construct a circRNA/microRNA (miRNA)/messenger RNA (mRNA) network involved in the progression and prognosis of GC. CircRNA DYRK1A_017, circRNA FLNA_118, miR-6512-3p, miR-6270-5p, and VCAN were identified as the key molecules in the hub regulatory axis. Dysregulation of this axis contributed to the cancer-associated signaling pathways (epithelial-mesenchymal transition [EMT], Nuclear factor kappa β-Tumor necrosis factor-α (NFκβ-TNFα) signaling, and angiogenesis) and aberrant immune microenvironment (infiltration by tumor associated macrophage, regulatory T cell, and mast cell). More importantly, the immunosuppressive tumor microenvironment may reveal the mechanism of novel circRNAs in tumors and serve as the target of immunotherapy.

## Introduction

As one of the most prevalent malignant tumors in both genders, gastric cancer (GC) is a major leading cause of cancer-related death worldwide in recent years. In 2018, there were an estimated more than 1 million GC cases globally (1, 2). Especially in East Asia, the incidence and mortality of GC remain high (3, 4). Unfortunately, since patients with early GC lack obvious and specific symptoms, the early diagnosed rate is low. Therefore, the 5-year survival rate with or without lymph node metastasis is <20 and 10%, respectively (5, 6). The WHO classification and tumor node metastasis (TNM) stage are generally utilized for hierarchical treatment. However, the prognosis among the patients with the same pathological stage varies, suggesting the important roles of gene heterogeneity and tumor microenvironment (TME) (7).

The tumor immune microenvironment is critical for the survival of patients (8, 9). Enormous effort is made to discover the immune targets and explore the optimal therapeutic interventions (10). In a cancer biological process, the immunosuppressive microenvironment is established to avoid an antitumor immune response. The mechanism that involves selective immune cell infiltration and immunosuppressive molecules is employed to escape from the immunological surveillance (11). The essential issues are proposed for the clinicians to uncover the status of the immune response, identify key immune elements, and integrate into the field of immunopathology (12). Compared with the routine strategies, immunotherapy (e.g., immune checkpoint inhibitors) shed light on patient management in various tumors. Several phase III trials were conducted to estimate the efficacy in advanced GC. However, in the randomized trial JAVELIN 300, Avelumab did not improve overall survival (OS) or post-progression survival (PFS) compared with chemotherapy (13). Similarly, as first-line or second-line therapy for advanced gastric cancer, Pembrolizumab was noninferior to chemotherapy in OS (14, 15). Despite some other trials that are currently ongoing (e.g., ATTRACTION-04, CheckMate-649, and KEYNOTE-859), these outcomes suggested an aberrant immune microenvironment in a GC tissue.

With the rapid development of molecular biology and high-throughput sequencing technology, non-coding RNA (ncRNA) is recognized in the process of tumor initiation and progression (16–18). Among them, circular RNA (circRNA) attracted attention for its regulatory function in various cellular, biological, and pathological processes (19, 20). Different from the linear RNA molecules, a circRNA holds a covalently closed continuous loop. This unique structure endows circRNA with resistance to RNase and being more stable than their linear counterparts. Most of the circRNAs are unable to translate into proteins; By sponging microRNA (miRNA), a circRNA regulates the transcriptional and translational behavior. Various circRNAs are identified as aberrantly expressed in the tumor tissues (18). For example, a circRNA CSNK1G3 interacts with miR-181 and promotes prostate cancer growth (21). A circRNA CCDC66 promotes colon cancer growth and metastasis (22). In terms of GC, only several studies have demonstrated the essential functions of circRNAs. Ding et al. (23) found that circ-DONSON facilitates GC growth and invasion *via* activation of transcription factor SOX4. CircNHSL1 was reported by Zhu et al. (24) that promotes the GC progression *via* miR-1306-3p/SIX1/vimentin axis. In addition, a circular RNA circ_HN1 was found as a modulator of the miR-302b/ROCK2 axis and facilitating GC progression (25). As an impact factor in GC, circRNA may pave the way for novel approaches for the management of patients (26–28). However, the association between a circRNA and TME of GC remains to be elucidated (29, 30).

In this study, we investigated the association between circRNAs and GC immune microenvironment. We constructed a ceRNA network and identified the hub circRNAs/miRNA/messenger RNA (mRNA) axis. Its underlying mechanism in the progression of GC and regulatory function in a tumor immune microenvironment were explored and discussed. Our findings demonstrated the regulatory mechanisms of circRNAs and contribute to the development of personalized immunotherapy for patients with GC.

## Materials and Methods

### Data Acquisition and Differential Expression Screening

In this study, we obtained a collection of expression data related to GC from The Cancer Genome Atlas (TCGA) database (https://www.cancer.gov/about-nci/organization/ccg/research/structural-genomics/tcga), including miRNA-seq, RNA-seq, and the corresponding clinical information (Supplementary Tables S1–S3). Based on the edger R software package (R Foundation for Statistical Computing, Vienna, Austria), the expression data with the corresponding clinical information and deduplication were analyzed to acquire the differentially expressed mRNAs and miRNAs (DEmRNAs and DEmiRNAs, respectively). Next, we used the |Log 2-fold change| ≥ 1.0 and *P* < 0.05 as cut-off conditions to screen mRNAs and miRNAs for further analysis. The relevant data obtained from TCGA are publicly available and do not require the approval of any local ethics committee. Besides, we searched gastric tumor circRNA expression microarray information from the public database Gene Expression Omnibus (GEO) (http://www.ncbi.nlm.nih.gov/geo/) (Supplementary Table S4). GSE141977 (data from the three primary GC tissues and three matched gastric normal tissues) was selected for further analysis. Limma package (version: 3.40.2) of R software was used to study the differential expression of circRNAs (DEcircRNAs). |Log 2-fold change| ≥ 0.9 and *P* < 0.05 were defined as the thresholds for the screening of differential expression of circRNAs. The probe sets without corresponding circBase ID (http://www.circbase.org) were discarded.

### Prediction of miRNA Binding Sites and Selected miRNA Pathway Enrichment

The miRNA binding sites of the circRNAs were predicted using the online website Cancer-Specific CircRNA (CSCD) (http://gb.whu.edu.cn/CSCD/). The candidate miRNAs were selected using the following criteria: upregulated circRNA paired with downregulated miRNA. On the contrary, the downregulated circRNAs were paired with upregulated miRNA. Further analyses were restricted to the intersection of the predicted miRNAs and DEmiRNAs from TCGA. Except for hsa_circ_0081069, 15 miRNAs without repetition were predicted by a surplus of five circRNAs in total. Using the miRPath V3.0, DEmiRNAs were enriched. Meanwhile, the structure diagram of potential circRNA was carried out through the web tool Cancer-Specific CircRNA (CSCD).

### Prediction of miRNA-mRNA Interactions

We used miRDB, TargetScan, and miRTarbase to predicate the interactions between the selected miRNAs and messenger RNAs (mRNAs). Likewise, matching paired miRNA-mRNA combinations were selected. In total, 151 overlappings predicted mRNAs were selected for further analysis.

### Gene Ontology (GO) and Kyoto Encyclopedia of Genes and Genomes (KEGG) Functional Enrichment Analyses

To realize the major functions of the above-mentioned 151 mRNAs, we conducted the gene function enrichment analysis, and Kyoto Encyclopedia of Genes and Genomes (KEGG) and gene ontology (GO) analysis were applied. To identify enriched KEGG and GO terms, Database for Annotation, Visualization, and Integrated Discovery (DAVID, https://david.ncifcrf.gov/) was employed.

### Construction of CircRNA-miRNA-mRNA Network

The network consisted of potential target genes to the miRNAs, potential target miRNAs to the differentially expressed circRNAs (DECs), and corresponding DECs. Cytoscape software (version 3.7.2, ISB, WA, USA) was utilized to visualize the circRNA-miRNA-mRNA network.

### Survival Analysis of the mRNAs and Identification of Hub Genes

To study the prognostic value of the DEmRNAs of the network, we used mRNA expression data and the clinical information downloaded from TCGA for survival analysis. The DEmRNAs with a log-rank *P* < 0.05 were considered as potential DEmRNAs and draw their survival curves through the R package “Survminer.” Ultimately, seven mRNAs revealed significant differences in the expression profile and survival outcomes synergistically and were selected as hub genes. Of which, STX1A and VCAN with a *p* < 0.01 were enrolled in further procedures. In addition, the clinical characteristics of the two mRNAs, such as age, gender, grade, stage, and TNM were analyzed. To compare the performance of STX1A and VCAN, the Gene Expression Profiling Interactive Analysis (GEPIA, http://gepia.cancer-pku.cn/) was processed.

### Gene Set Enrichment Analysis (GSEA) and Immune Infiltration Analysis

As a computational method, gene set enrichment analysis (GSEA) is considered for the interpretation of gene expression data. We obtained the pathways that were significantly related to the central gene with GSEA analysis, and the cut-off value was set to *p* < 0.05. The immunohistochemical staining results were obtained from the Human Protein Atlas. An immune infiltration analysis was performed using TIMER (http://cistrome.dfci.harvard.edu/TIMER/) and TISIDB (http://cis.hku.hk/TISIDB/).

### Statistical Analysis

All the analyses were performed using R (version3.6.1; https://www.r-project.org/). All the statistical comparisons between the two groups were performed with the Student's *t*-test, and *p* < 0.05 was considered significant.

## Results

### Identification of DECs

**Figure 1:**
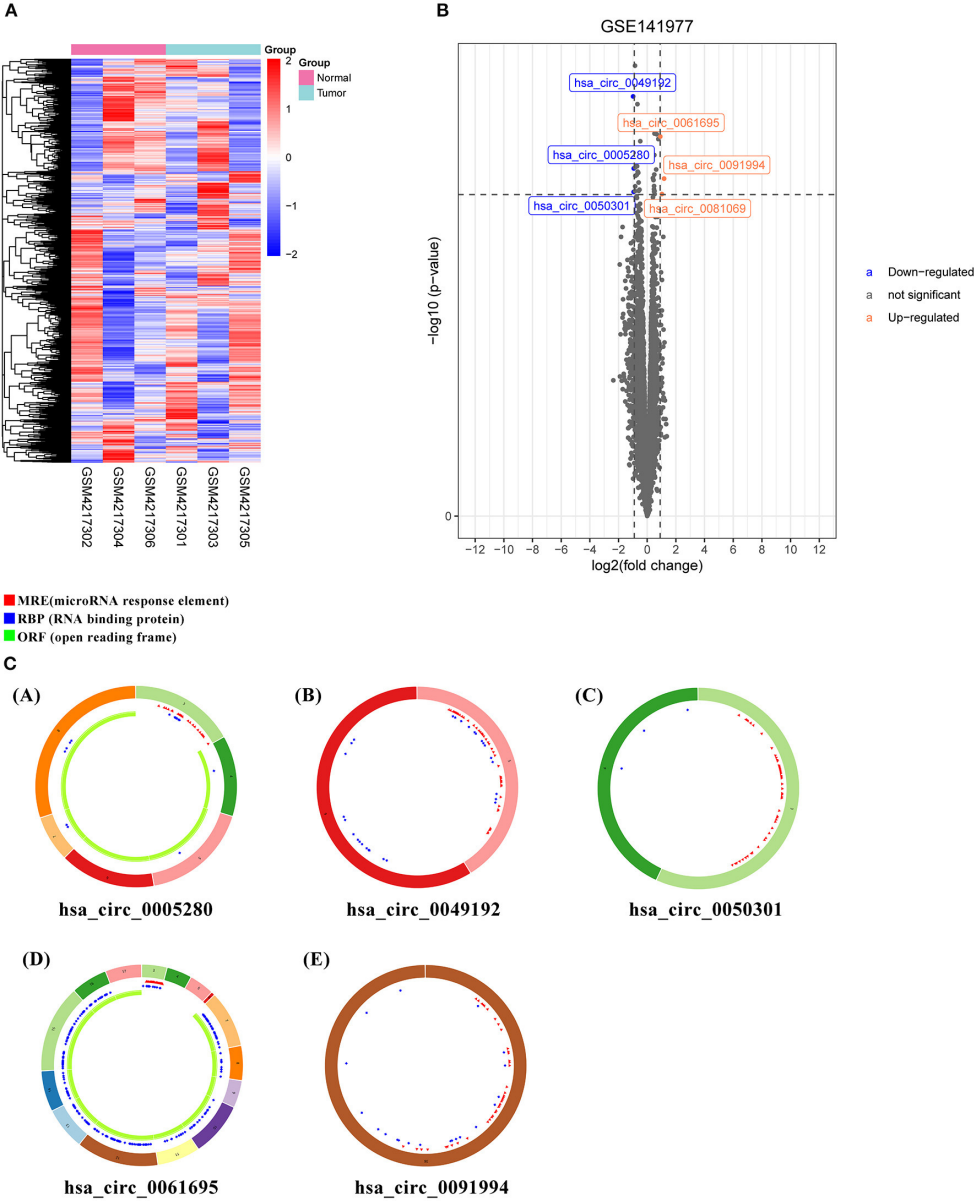
Differently expressed circular RNAs (DECs) and predicted CircRNA structure. **(A)** Heatmap of top six DECs. **(B)** Volcano plot of DECs. **(C)** Predicted CircRNA structure of the top six DECs. Each arc with a numeric ID depicts one exon. The red spots represent microRNA (miRNA) response elements (MRE), the blue spots represent RNA binding protein (RBP), and the green curves represent open reading frame (ORF).

The microarray GSE141977 from the GEO dataset was contained in the study. After normalization of batch effect, six circRNAs were defined as DECs between tumor and normal tissue: hsa_circ_0005280, hsa_circ_0049192, hsa_circ_0050301, hsa_circ_0061695, hsa_circ_0081069, and hsa_circ_0091994 (Supplementary Table S5). Among which, hsa_circ_0005280, hsa_circ_0049192, and hsa_circ_0050301 were downregulated, while hsa_circ_0061695, hsa_circ_0081069, and hsa_circ_0091994 were upregulated in tumor. The circRNAs were displayed in the heatmap and volcano map (Figures 1A,B).

### Identification of Target miRNAs and Target mRNAs

**Figure 2:**
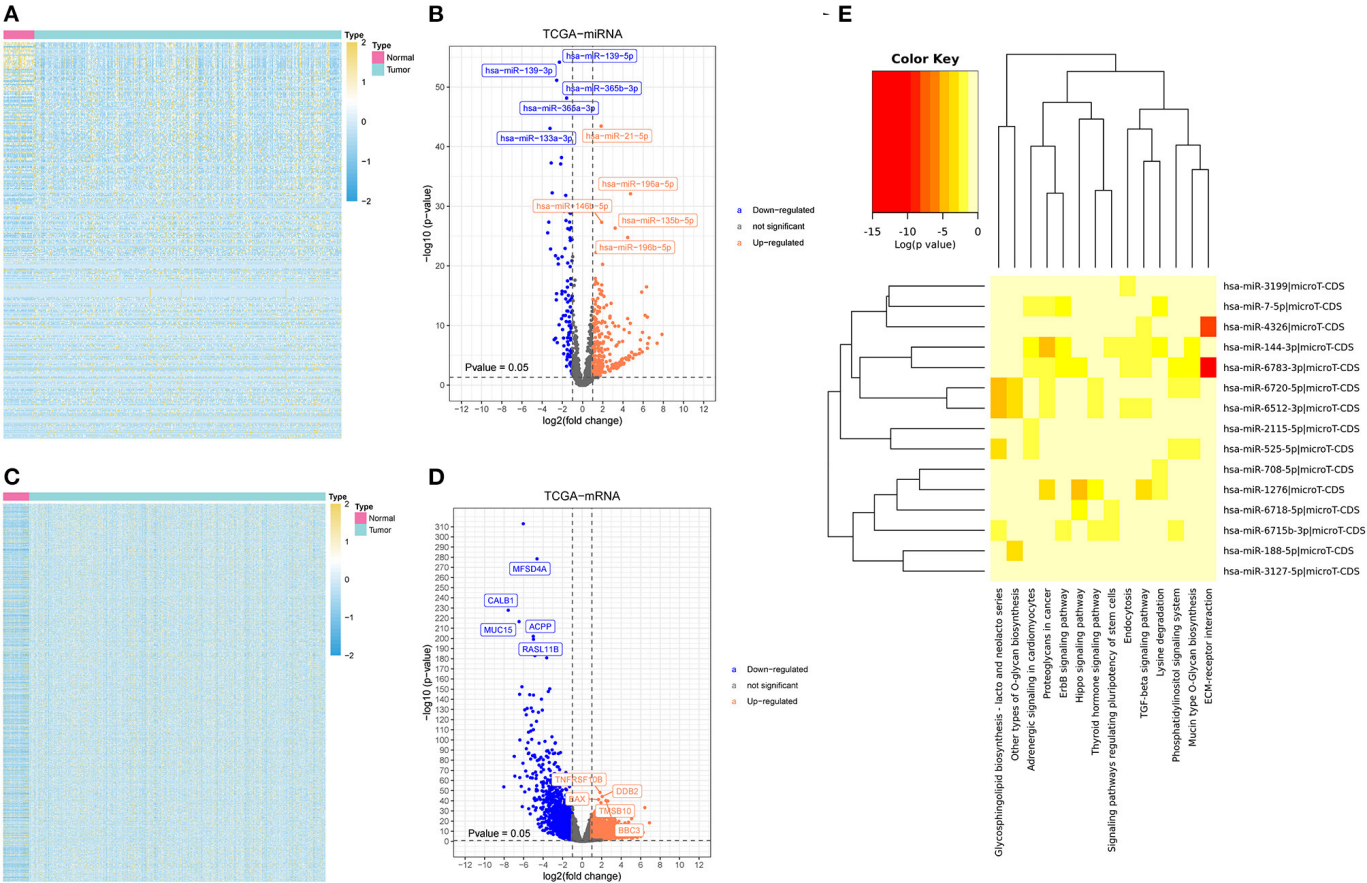
Differently expressed miRNAs and messenger RNAs (mRNAs) (DEmiRNAs and DEmRNAs, respectively) and the possible associated signal pathways **(A)** a heatmap of DEmiRNAs. **(B)** A volcano plot of DEmiRNAs. **(C)** A heatmap of DEmRNAs. **(D)** A volcano plot of DEmRNAs. **(E)** The possible signal pathways in which 15 miRNAs may be involved.

To identify miRNA response element (MRE) and target miRNA, the above-mentioned DECs were predicted by the Cancer-Specific CircRNA Database (CSCD), among which the five DECs can be identified. The pattern map of five DECs, namely MRE, RNA binding protein, and open reading frame information, was obtained from the CSCD (Figure 1C). From the TCGA database, we obtained the quantitative data of miRNA and mRNA expression in 270 GC samples *via* sequencing (miRNA-seq and mRNA-seq). R software was used to screen the DEmiRNAs and DEmRNAs extracted from TCGA. After the intersection of the above-predicted miRNAs and DEmiRNAs, 15 miRNAs were identified as target miRNAs (Figures 2A,B; Supplementary Table S5). Subsequently, the MiRDB database, the TargetScan database, and the DIANA database were utilized to predict the target mRNA of these 15 miRNAs, respectively. In total, 151 eligible genes (mRNAs) were selected as the final target genes by the intersection with DEmRNAs (Figures 2C,D; Supplementary Table S6).

### Functional Annotation of the Target miRNAs and Target mRNAs

A software named as DIANA-MiRPath V3.0 was utilized to investigate the possible signal pathways in which 15 miRNAs may be involved. The results indicated that several miRNAs were closely related to the tumor-associated signal pathways (Figure 2E). To explore the understanding of the biological relevance of these 151 target genes, KEGG and GO analyses were conducted. According to the results of DAVID, the top enriched biological process, cellular components, and molecular function terms were: regulation of transcription from RNA polymerase II promoter, plasma membrane, and transcription factor activity, respectively (Supplementary Figure S1).

### Construction of the CeRNA Network

**Figure 3:**
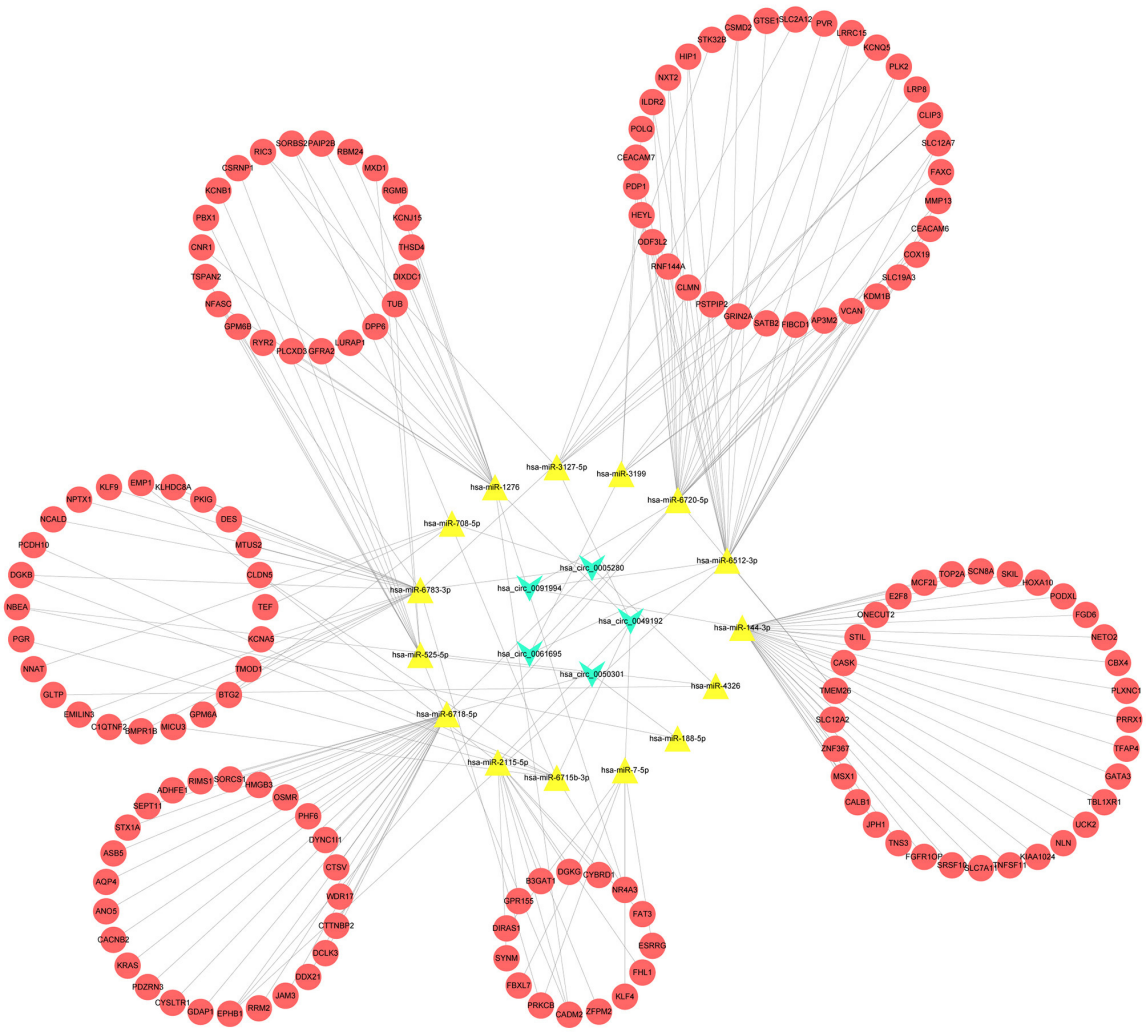
A competing endogenous RNA (CeRNA) regulation network constructed by the circRNAs, 15 miRNAs, and 151 target mRNAs.

After multiple filters, five circRNAs, 15miRNAs, and 151 target mRNAs were screened out for subsequent investigation. The ceRNA (circRNA/miRNA/mRNA) network was established by a comprehensive consideration of the circRNA/miRNA interaction and miRNA/mRNA interaction, and initially displayed the overview of the ceRNA regulation network (Figure 3). The detailed matching information is shown in Supplementary Table S6.

### Cox Regression Analysis and Clinical Correlation Analysis

**Figure 4:**
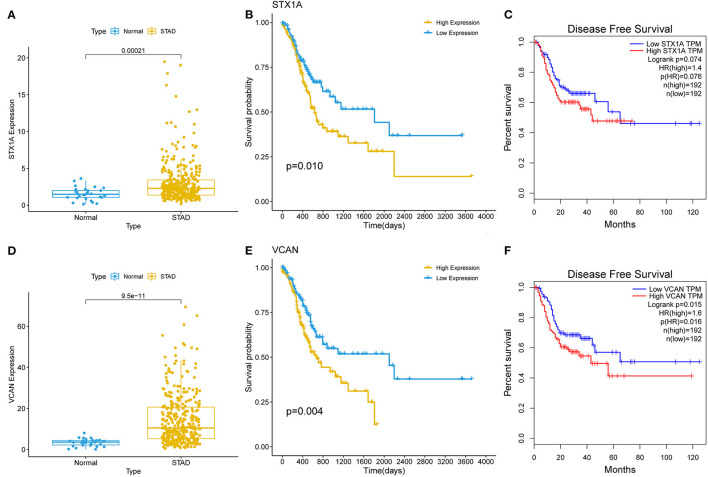
The survival analyses of two target genes (VCAN and STX1A). **(A)** Kalpan–Meier (K–M) plot of STX1A. **(B)** Expression of STX1A between gastric cancer (GC) and normal tissues. **(C)** Disease-free survival of STX1A. **(E)** K–M plot of VCAN. **(D)** Expression of VCAN between the GC and normal tissues. **(F)** Disease-free survival of VCAN. The relationship between a gene and survival was analyzed through the K–M curve which was evaluated by log-rank test.

**Figure 5:**
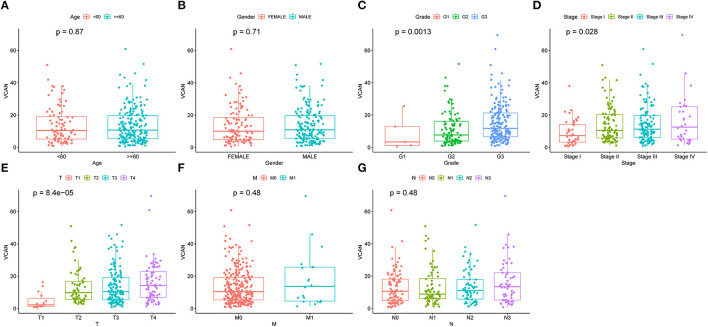
An Upregulated VCAN is associated with the GC progression. The clinical characteristics, including **(A)** age. **(B)** Gender. **(C)** Grade. **(D)** Stage. **(E)** T stage. **(F)** M stage, and **(G)** N stage of VCAN. Of which, upregulated VCAN indicates higher pathological grade (*p* = 0.0013), stage (*p* = 0.028), and T stage (*p* = 8.4e-5). The relationship between the VCAN and clinical characteristics was analyzed by Wilcox's of R software.

A survival analysis of the 151 target genes was conducted, and the target genes significantly associated with the OS were shown in Supplementary Table S7. Seven genes were holding the same tendency between the differentially expressed analysis and survival analysis. Among them, the two target genes (VCAN and STX1A) with a *P* < 0.01 were selected for further analysis (Figure 4; Supplementary Figure S2). Particularly, VCAN was significantly associated with disease-free survival (*P* = 0.016) while STX1A was not (*P* = 0.076). Based on Wilcox's of R software, the association between each subset of age, gender, grade, stage, and TNM with the two candidate genes was analyzed. We found that VCAN was significantly associated with grade (*P* = 0.0013), stage (*P* = 0.028), and T stage (*P* = 8.4 × 10^−5^; Figure 5), while STX1A was significantly associated with age (*P* = 0.035) and grade (*P* = 0.00086; Supplementary Figure S3).

### Construction of Regulatory Axis

**Figure 6:**
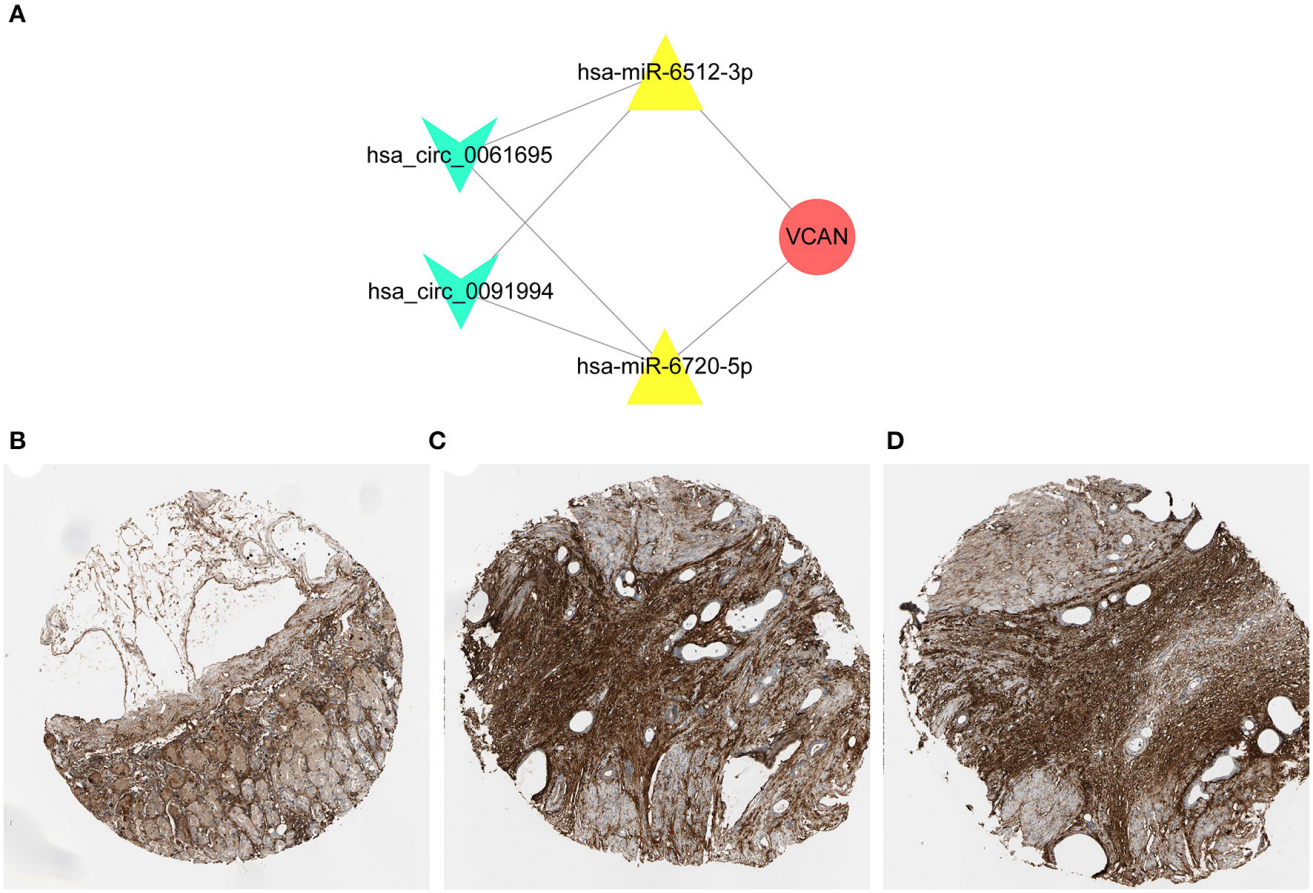
Construction of regulatory axis. **(A)** The key axis in the circRNA-miRNA-mRNA network of GC was constructed by hsa_circ_0061695, hsa_circ_0091994, hsa-miR-6512-3p, hsa-miR-6720-5p, and VCAN. **(B–D)** The immunohistochemical staining results indicated that a higher expression level of VCAN was confirmed in the tumor tissues. The samples of normal tissue **(B)** and two tumor tissues **(C,D)** were obtained from one patient. Concrete information at https://www.proteinatlas.org/.

Based on the previous analysis, we identified VCAN as the star hub gene in the regulatory ceRNA network and the hub regulatory axis was defined as hsa_circ_0061695&hsa_circ_0091994/hsa-miR-6512-3p&hsa-miR-6720-5p/VCAN (Figure 6A). These two novel circRNAs were first identified as GC promoters. Furthermore, we employed the Human Protein Atlas for subsequent verification. The immunohistochemical staining results indicated that a higher expression level of VCAN was confirmed in the tumor tissues (Figures 6B–D).

### Gene Set Enrichment Analysis

**Figure 7:**
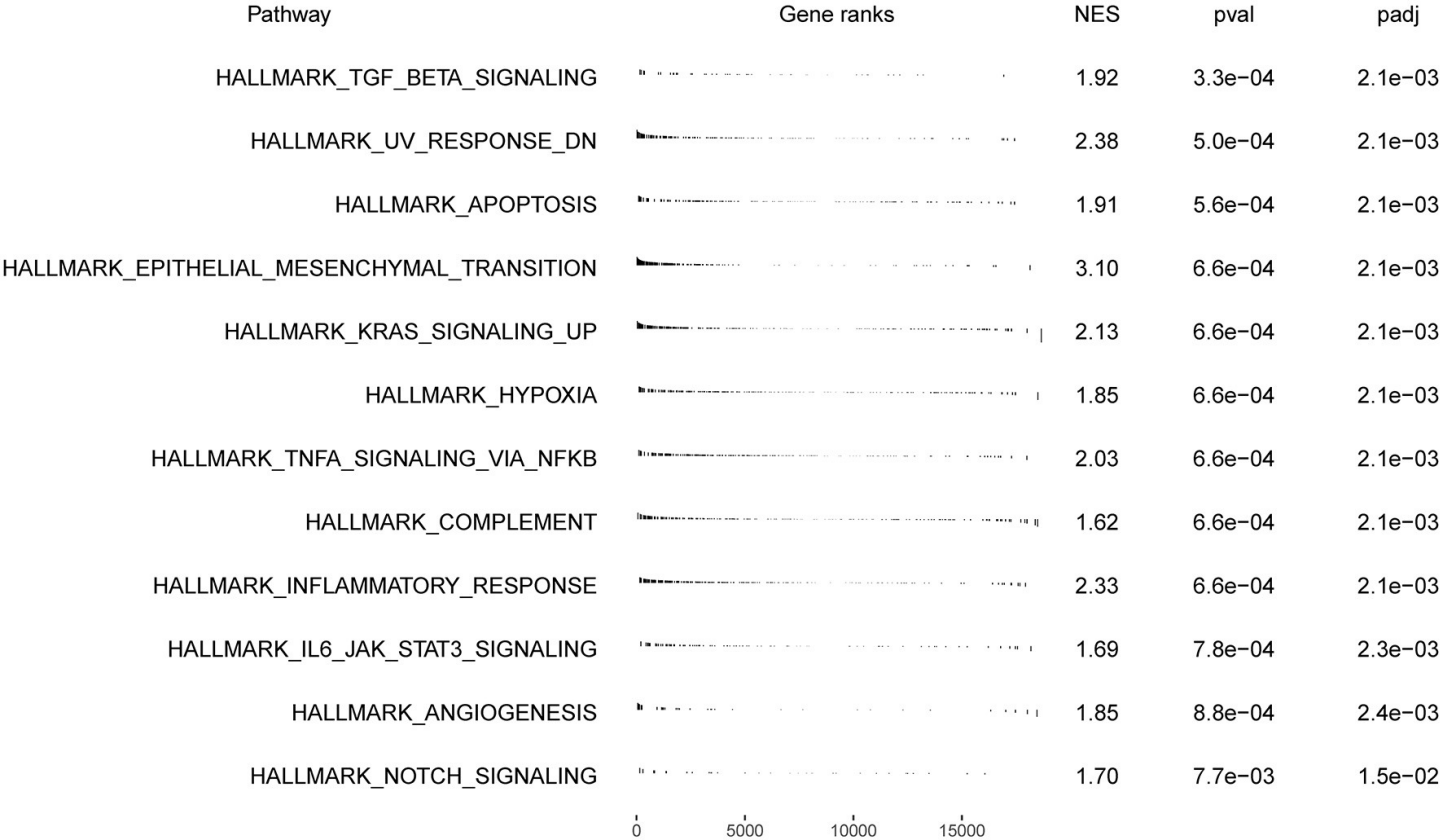
Biological relevance between the VCAN and GC initiation and progression.

To investigate the biological relevance between the hub regulatory axis and GC initiation and progression, a GSEA was conducted. The result indicated that most signal pathways enriched were mainly associated with the regulation of GC tumorigenesis and progression process, such as TGF β signaling, epithelial-mesenchymal transition (EMT), KRAS signaling, hypoxia, TNFα-NFKβ signaling, inflammatory response, IL6-JAC-STAT signaling, angiogenesis, and NOTCH signaling (Figure 7).

### The Immune Landscape of the Regulatory Axis

**Figure 8:**
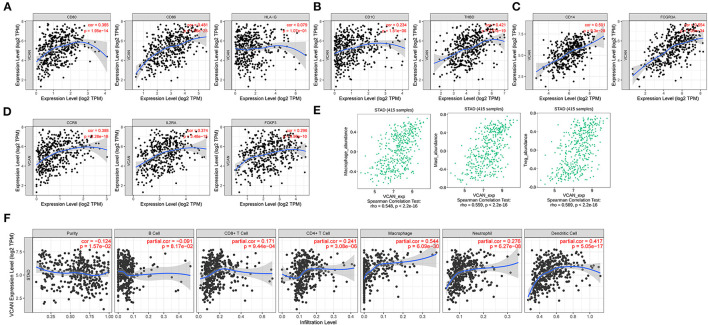
Immune infiltration of VCAN. **(A–C,E)** Biomarkers of **(A)** dendritic cell, **(B)** monocyte, **(C)** tumor-associated macrophage (TAM), and **(E)** regulatory T cell (Treg). **(D)** VCAN expression is associated with the macrophage, mast cell, and Treg infiltration. **(F)** Major immune cells infiltration in the GC tissues is correlated with the VCAN expression.

The importance of the immune system in the tumor process is now recognized. The correlation between the regulatory axis and immune infiltration was evaluated. The results indicated that the expression levels, including CD1C, THBD, CD14, FCGR3A, CD80, CD86, HLA-G, CCR8, IL2RA, and FOXP3, increased stepwise with VCAN expression level rising. The increased expression level of CCR8, interleukin 2 receptor alpha-chain (IL2RA), and Foxp3 indicate regulatory T cell (Treg) infiltration and contribute to the immunosuppression of T cells against cancer (31). The upregulated CD80 and CD86 enhance the suppressive function of the induced Treg cells (32). Moreover, the tumor-associated macrophagocytes, mast cells, regulatory T cells, dendritic cells were positively associated with the upregulated axis (Figure 8, all *P* < 0.05). In addition, we analyzed the impacts of VCAN copy number variation on several infiltration levels of the pivotal immune cell subtypes. The arm-level deletion is the most obvious variation type and significantly reduce B cell (*p* < 0.05), CD8+T cell (*p* < 0.001), CD4+T cell (*p* < 0.001), macrophage (*p* < 0.001), neutrophil (*p* < 0.001), and dendritic cell (*p* < 0.001). Deep deletion of VCAN also exhibited the suppressor effect on immune system, with a low infiltration level of CD8+T cell (*p* < 0.05), macrophage (*p* < 0.05), neutrophil (*p* < 0.01), and dendritic cell (*p* < 0.01; Supplementary Figure S4A). Spearman's correlations between the expression of VCAN and tumor-infiltrating lymphocytes (*Y*-axis) across human cancers (*X*-axis) were also demonstrated (Supplementary Figure S4B). These results indicated that the regulatory axis mediated immunosuppressive microenvironment contributed to the GC progression. The molecular mechanisms are worthy of further discussion.

## Discussion

GC is a digestive malignancy with high incidence and mortality around the world. With a diagnostic rate of early GC of <10%, most of the patients were diagnosed at an advanced stage and succumb to a worse prognosis and quality of life. About 50–70% of the patients with advanced GC suffered from recurrence and resulted in an unsatisfied 5-year survival rate after routine treatment (33). Because of gene heterogeneity, not everyone was benefitted from surgery or chemotherapy. Immunotherapy may be a promising option. In this scenario, understanding the immune microenvironment and exploring the regulatory factors of GC are urgently expected. Herein, we determined the key circRNA-miRNA-mRNA axis of GC. We found that hsa_circ_0061695, hsa_circ_0091994, hsa-miR-6512-3p, hsa-miR-6720-5p, and VCAN are the key nodes in the network. Among these, hsa_circ_0061695 and hsa_circ_0091994 were the first found associated with tumor progression. To better understand the mechanism, we performed the GO and KEGG analysis and GESA. We eventually extracted the infiltrated immune cell subsets that contribute to an immunosuppressive microenvironment regulated by the axis.

In recent years, emerging evidence has found abnormalities in the molecular mechanisms of GC, such as the abnormal regulators in the gene expression. The circRNAs were traditionally recognized as meaningless byproducts (34). Recent studies have proved circRNAs as an abundant and diverse type of RNA associated with various cellular processes and involved in the initiation and development of substantial diseases, such as cancer (35). The circRNAs that contain one or more MREs that could act as miRNA sponges and negatively modulate the miRNA activity. Therefore, the inhibitory effect on target genes by miRNAs is attenuated by the circRNAs. Though several circRNAs are found to be participating in the immune responses (36), such as binding and suppressing the kinase PKR (37); the immune regulatory function of circRNAs is awaiting deeper exploration (38).

In this study, we first described the regulatory function of hsa_circDYRK1A_017 (hsa_circ_0061695). Hsa_circFLNA_118 (hsa_circ_0091994) was recently reported as a cancer promotor in accordance with our results (39). We constructed the novel regulatory axis significantly correlated to the clinical outcomes. The results found that hsa_circDYRK1A_017 locates in +strand of chr21 (40), while hsa_circFLNA_118 locates in a strand of chrX (41). These circRNAs contribute to the malignant biological process in GC and shed light on the potent applications for early detection as well as novel therapeutic targets (28). Correspondingly, miR-6512-3p and miR-6270-5p were downregulated in the GC tissues and pointed out to be associated with skin reveals (42) and fibrosis (43). Herein, the circRNAs could modulate the GC progression through sponging miR-6512-3p and miR-6270-5p. Several studies focused on the impacts of VCAN on the GC initiation and progression (44, 45). Ye et al. reported that the upregulation of VCAN promotes GC proliferation, invasion, and migration (46). In another study, lncRNA VCAN-AS1 contributed to the progression of GC through regulation of the p53 expression (47). VCAN could serve as the novel therapeutic target or biomarker for GC (48–51). These results revealed the remarkable function of the ceRNA axis in tumorigenesis and the progression of GC.

In recent years, though great efforts were made to decipher the molecular basis of the heterogeneity, and significant progress has been made to understand the intrinsic pathogenesis of GC, the treatment made slow progression. Fluoropyrimidine and platinum-based chemotherapy as the first-line therapy and paclitaxel agents as second-line therapy are the standard treatment for advanced GC; however, the prognosis remained dismal (52–54). Involved in many cell biological behaviors, including cell adhesion, proliferation, migration, extracellular matrix assembly, and survival, a tumor immunosuppressive microenvironment plays an immense role in cell biology (55). The immunosuppressive molecules (e.g., PD-L1 and CTLA-4) and cells targeted therapies have revealed the prominent anticancer properties and suggested the possible approach to immunotherapy in regulating the circRNAs/miRNA/mRNA axis. A ceRNA was identified to be associated with the growth of tumors. However, the underlying mechanism remained obscure. In this study, we found that the hub ceRNA axis significantly promotes tumor progression *via* TME and immune cell reprogramming. The elevated CD1c^+^ dendritic cells were reported as the prognostic indicator for GC (56). Defected DCs CCR8, a chemokine receptor mainly expressed on the Treg cells plays a crucial role in the CCR8^+^ Treg-mediated immunosuppression and is uniquely upregulated in human tumor-resident Treg (31, 57). Another study indicated that Foxp3 in the tumor-infiltrating Treg cells serves as the immunosuppressive molecule to T-cell proliferation (58). HLA-G inhibits the function of immunocompetent cells, including natural killer (NK) and T cells, and represents a strategy employed by the tumors for the resistance to immune rejection (59). An immune cell infiltration analysis further confirms the immunosuppressive microenvironment. Apart from TAM and Treg, the upregulated mast cell is recognized. The recruited and co-localized Treg and mast cells in the tumor regions represented resistance to anti-PD-1 treatment (60). Overall, our data reinforced the association between the immune cell subtypes infiltration and VCAN expression. These results suggest that the circRNA/miRNA/mRNA axis may regulate the GC progression *via* tumor immune microenvironment and serve as a potential target for immunotherapy against GC.

## Data Availability Statement

The datasets presented in this study can be found in online repositories. The names of the repository/repositories and accession number(s) can be found in the article/Supplementary Material.

## Author Contributions

YD, WG, and SL designed the research. XL, ZL, and PL collected the data. SA, FS, and QH performed the data analysis. XL wrote the manuscript. XX, WG, and SL reviewed the manuscript. All the authors reviewed and approved the manuscript.

## Funding

This work was supported by the National Natural Science Foundation of China (81602103 and 82172645), the Natural Science Foundation of Jiangsu Province (BK20200052), and the Clinical Trials from the Affiliated Drum Tower Hospital, Medical School of Nanjing University (2021-LCYJ-MS-09 and 2021-LCYJ-PY-17).

## Conflict of Interest

The authors declare that the research was conducted in the absence of any commercial or financial relationships that could be construed as a potential conflict of interest.

## Publisher's Note

All claims expressed in this article are solely those of the authors and do not necessarily represent those of their affiliated organizations, or those of the publisher, the editors and the reviewers. Any product that may be evaluated in this article, or claim that may be made by its manufacturer, is not guaranteed or endorsed by the publisher.
