# Supplementary material for: Novel CircRNAs in Hub ceRNA Axis Regulate Gastric Cancer Prognosis and Microenvironment
Source: Front Med (Lausanne). 2021 Nov 8;8:771206. doi: 10.3389/fmed.2021.771206 (PMC8606568; doi:10.3389/fmed.2021.771206)
Supplement: Supplementary file 5 [file Data_Sheet_1.docx]

**Supplementary Information**

**Supplementary Tables**

**Table S5**. Information of DEcircRNAs.

| CircRNA_ID | Position | Strand | Best transcript | Gene symbol | Regulation |
| --- | --- | --- | --- | --- | --- |
| hsa_circ_0005280 | chr2: 168869143-168931741 | - | NM_013233 | STK39 | Down |
| hsa_circ_0049192 | chr19: 10246411-10246963 | - | NM_001130823 | DNMT1 | Down |
| hsa_circ_0050301 | chr19: 22255610-22256366 | + | NM_033468 | ZNF257 | Down |
| hsa_circ_0061695 | chr21: 38792600-38878526 | + | NM_101395 | DYRK1A | Up |
| hsa_circ_0081069 | chr7: 94027693-94037203 | + | NM_000089 | COL1A2 | Up |
| hsa_circ_0091994 | chrX: 153590346-153590516 | - | NM_001110556 | FLNA | Up |

**Table S6** CeRNA matched network**.**

| CircRNA | miRNA | mRNA |
| --- | --- | --- |
| hsa_circ_0005280 | hsa-miR-4326 | GLTP、NBEA |
|  | hsa-miR-6718-5p | SORCS1、HMGB3、DYNC1I1、ASB5、CTTNBP2、RIMS1、PHF6、EPHB1、KRAS ADHFE1、CYSLTR1、OSMR、SEPT11、  RRM2、JAM3、CTSV、ANO5、STX1A、GDAP1、CACNB2、AQP4、WDR17、PAZRN3、DCLK3、DDX21 |
|  | hsa-miR-6783-3p | KCNA5、MTUS2、EMP1、DGKB、NPTX1、BTG2、TMOD1、DES、EMILIN3、C1QTNF2、CLIP3、KLF9、NFASC、BMPR1B、KLHDC8A、PRKCB、NCALD、PKIG |
|  | hsa-miR-708-5p | GPM6A、NNAT、TEF |
| hsa_circ_0049192 | hsa-miR-1276 | DPP6、CADM2、NR4A3、KCNJ15、RBM24、RYR2、SORBS2、PLCXD3、THSD4、RIC3、NFASC、DIXDC1、PAIP2B、LURAP1、CNR1、RGMB |
|  | hsa-miR-2115-5p | FAT3、EMP1、CADM2、FHL1、SYNM、DGKG、B3GAT1、GPR155、CYBRD1、ZFPM2 |
|  | hsa-miR-3127-5p | CLIP3、FAXC、RIC3、KCNQ5、GRIN2A、SLC2A12、STK32B |
|  | hsa-miR-6715b-3p | DGKB、NR4A3、PGR、PCDH10、SORBS2、MICU3、CLDN5 |
|  | hsa-miR-7-5p | ESRRG、NR4A3、KLF4、DIRAS1、FBXL7、PRKCB |
| hsa_circ_0050301 | hsa-miR-188-5p | NBEA |
|  | hsa-miR-6718-5p | 同上 |
|  | hsa-miR-525-5p | CADM2、KCNB1、GPM6B、GFRA2、CSRNP1、TSPAN2、NFASC、MXD1、TUB、PBX1 |
| hsa_circ_0061695 | hsa-miR-3199 | COX19、AP3M2、PDP1、SATB2、POLQ、LRP8 |
|  | hsa-miR-6512-3p | HIP1、KDM1B、RNF144A、PLK2、PSTPIP2、CLMN、NXT2、EPHB1、ODF3L2、ILDR2、SLC12A7、HEYL、CEACAM7、CEACAM6、SLC19A3、CSMD2、VCAN、GTSE1、TMEM26、LRRC15、FIBCD1、MMP13 |
|  | hsa-miR-6720-5p | HIP1、KDM1B、RNF144A、PLK2、PSTPIP2、CLMN、NXT2、EPHB1、ODF3L2、ILDR2、SLC12A7、PVR、HEYL、CEACAM7、CEACAM6、SLC19A3、CSMD2、VCAN、TMEM26、LRRC15、FIBCD1、MMP13 |
| hsa_circ_0091994 | hsa-miR-144-3p | PLXNC1、SRSF10、FGD6、TNS3、FGFR10P、SLC12A2、NLN、CBX4、SKIL、TBL1XR1、UCK2、GATA3、SLC7A11、MCF2L、TFAP4、CAASK、E2F8、MSX1、PRRX1、JPH1、SCN8A、KIAA1024、PODXL、ONECUT2、ZNF367、NET02、STIL、TNFSF11、TOP2A、HOXA10、CALB1 |
|  | hsa-miR-6512-3p | PLXNC1、SRSF10、FGD6、TNS3、FGFR10P、SLC12A2、NLN、CBX4、SKIL、TBL1XR1、UCK2、GATA3、SLC7A11、MCF2L、TFAP4、CAASK、E2F8、MSX1、PRRX1、JPH1、SCN8A、KIAA1024、PODXL、ONECUT2、ZNF367、NET02、STIL、TNFSF11、TOP2A、HOXA10、CALB1 |
|  | hsa-miR-6720-5p | PLXNC1、SRSF10、FGD6、TNS3、FGFR10P、SLC12A2、NLN、CBX4、SKIL、TBL1XR1、UCK2、GATA3、SLC7A11、MCF2L、TFAP4、CAASK、E2F8、MSX1、PRRX1、JPH1、SCN8A、KIAA1024、PODXL、ONECUT2、ZNF367、NET02、STIL、TNFSF11、TOP2A、HOXA10、CALB1 |

**Table S7** Overall survival of genes with significant differences by Kaplan-Meier analysis (P-value < 0.05).

| Gene | KM (P-value) |
| --- | --- |
| DIRAS1 | 0.0249486855415721 |
| FBXL7 | 0.0314521388569285 |
| FGD6 | 0.0425869659803041 |
| E2F8 | 0.0379079982952086 |
| STIL | 0.0455855701681931 |
| HOXA10 | 0.0448466485988398 |
| GFRA2 | 0.00174840235153517 |
| TUB | 0.036325620105505 |
| PLCXD3 | 0.00964351513495809 |
| FAT3 | 0.00294424041107955 |
| ZFPM2 | 0.0452781582809788 |
| POLQ | 0.00610860843196825 |
| ILDR2 | 0.0460746291612633 |
| CSMD2 | 0.0216138906078336 |
| VCAN | 0.00354326371382874 |
| TMEM26 | 0.0437627514537562 |
| PGR | 0.0407167889387792 |
| CLDN5 | 0.0206328765207536 |
| DYNC1I1 | 0.000381540195364072 |
| ADHFE1 | 0.0429149626185272 |
| SEPT11 | 0.0168674329661747 |
| JAM3 | 0.0083512374302438 |
| STX1A | 0.00969130926476369 |
| PDZRN3 | 0.0253011596589933 |
| C1QTNF2 | 0.00427908035064717 |
| KLF9 | 0.0302769284117789 |
| BMPR1B | 0.0439333274981476 |
| PKIG | 0.046285838035704 |

**Supplementary Figures.**

**Figure S1**. Functional analysis of 151 target mRNAs by Kyoto Encyclopedia of Genes and Genomes (KEGG) and Gene Ontology (GO) analysis. (A) Biological process (B) Cellular component (C) Molecular function (D) KEGG pathways.

**Figure S2**. Expression profile and survival analysis of other 5 hub mRNAs.

**Figure S3**. Clinical characteristics including (A) age. (B) Gender. (C) Grade. (D) Stage. (E) T stage. (F) M stage, and (G) N stage of STX1A.

**Figure S4**. (A) Copy number variation of VCAN influence immune cells infiltration. (B) Spearman correlations between expression of VCAN and tumor-infiltrating lymphocytes (Y-axis) across human cancers (X-axis).
