# Supplementary figures and images for: Novel CircRNAs in Hub ceRNA Axis Regulate Gastric Cancer Prognosis and Microenvironment
Source: Front Med (Lausanne). 2021 Nov 8;8:771206. doi: 10.3389/fmed.2021.771206 (PMC8606568; doi:10.3389/fmed.2021.771206)

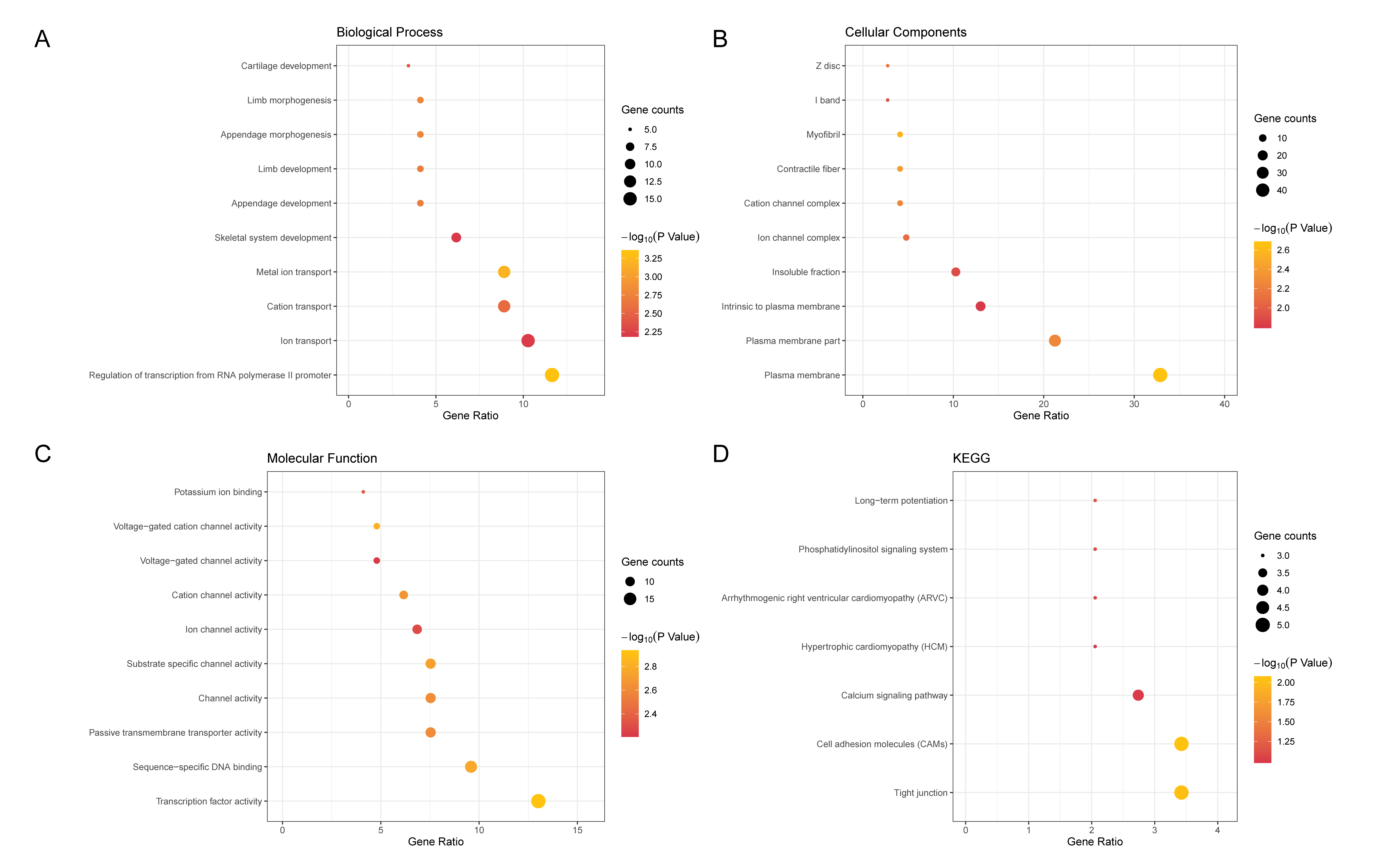

Supplement: Supplementary file 6 [file Image_1.JPEG]

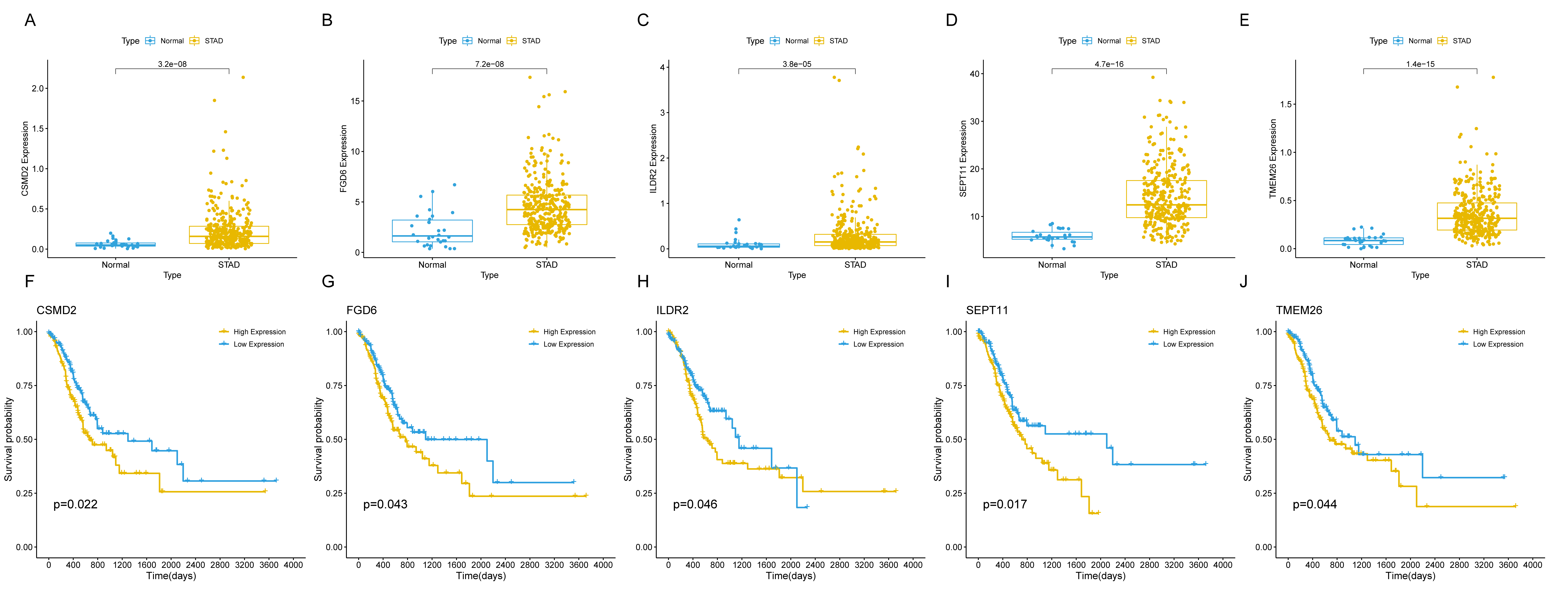

Supplement: Supplementary file 7 [file Image_2.JPEG]

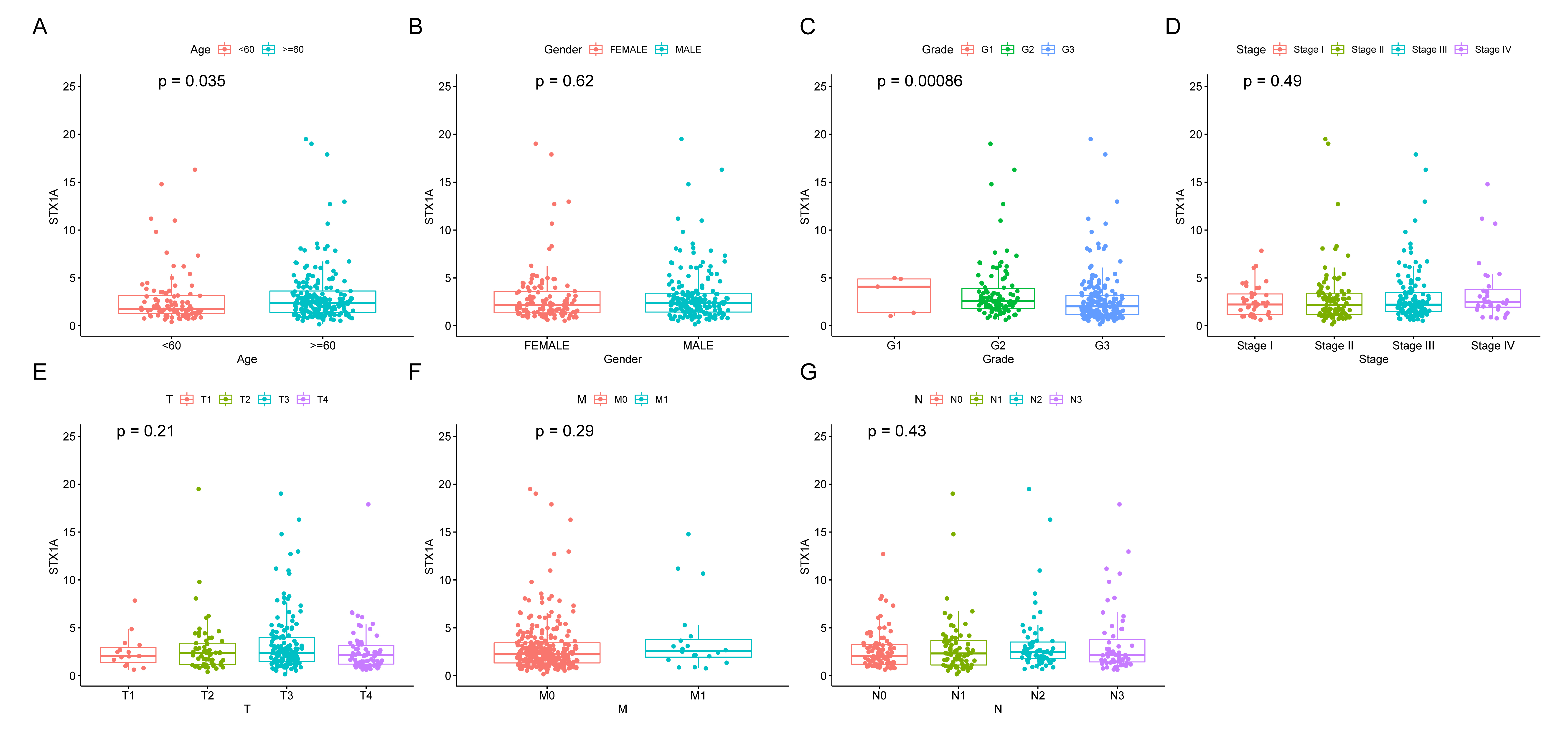

Supplement: Supplementary file 8 [file Image_3.JPEG]

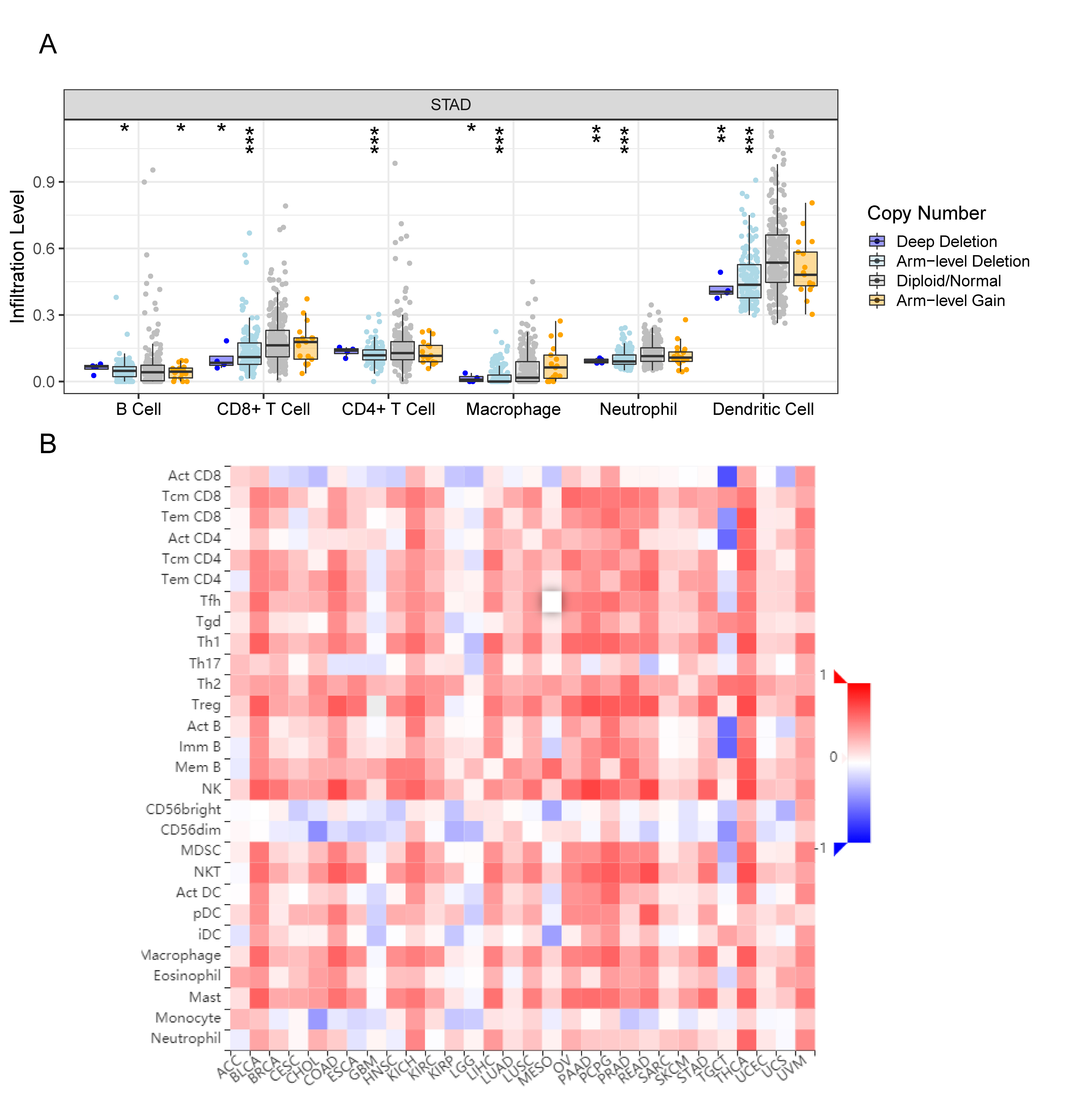

Supplement: Supplementary file 9 [file Image_4.JPEG]
